# Socio-demographic and clinical characteristics of re-presentation to an Australian inner-city emergency department: implications for service delivery

**DOI:** 10.1186/1471-2458-7-320

**Published:** 2007-11-10

**Authors:** Gaye Moore, Marie Gerdtz, Elizabeth Manias, Graham Hepworth, Andrew Dent

**Affiliations:** 1 The School of Nursing, The University of Melbourne, Parkville, Melbourne, Australia; 2 Emergency Department, St Vincent's Hospital Melbourne, Victoria Parade, Melbourne, Australia; 3 Statistical Consulting Centre, The University of Melbourne, Parkville, Melbourne, Australia

## Abstract

**Background:**

People who have complex health care needs frequently access emergency departments for treatment of acute illness and injury. In particular, evidence suggests that those who are homeless, or suffer mental illness, or have a history of substance misuse, are often repeat users of emergency departments. The aim of this study was to describe the socio-demographic and clinical characteristics of emergency department re-presentations. Re-presentation was defined as a return visit to the same emergency department within 28 days of discharge from hospital.

**Methods:**

A retrospective cohort study was conducted of emergency department presentations occurring over a 24-month period to an Australian inner-city hospital. Characteristics were examined for their influence on the binary outcome of re-presentation within 28 days of discharge using logistic regression with the variable patient fitted as a random effect.

**Results:**

From 64,147 presentations to the emergency department the re-presentation rate was 18.0% (n = 11,559) of visits and 14.4% (5,894/40,942) of all patients. Median time to re-presentation was 6 days, with more than half occurring within one week of discharge (60.8%; n = 6,873), and more than three-quarters within two weeks (80.9%; n = 9,151). The odds of re-presentation increased three-fold for people who were homeless compared to those living in stable accommodation (adjusted OR 3.09; 95% CI, 2.83 to 3.36). Similarly, the odds of re-presentation were significantly higher for patients receiving a government pension compared to those who did not (adjusted OR 1.73; 95% CI, 1.63 to 1.84), patients who left part-way through treatment compared to those who completed treatment and were discharged home (adjusted OR 1.64; 95% CI, 1.36 to 1.99), and those discharged to a residential-care facility compared to those who were discharged home (adjusted OR 1.46: 95% CI, 1.03 to 2.06).

**Conclusion:**

Emergency department re-presentation rates cluster around one week after discharge and rapidly decrease thereafter. Housing status and being a recipient of a government pension are the most significant risk factors. Early identification and appropriate referrals for those patients who are at risk of emergency department re-presentation will assist in the development of targeted strategies to improve health service delivery to this vulnerable group.

## Background

A relatively small proportion of frequent emergency department (ED) users continue to account for a significant number of all ED visits and consume a disproportionate amount of resources [1-6]. Although not the principal cause of overcrowding, frequent ED attenders may exacerbate the problems by increasing demand for acute care services in a climate of limited resources [7,8].

The literature describes well the characteristics of frequent ED users [1,2,9-20]. This group comprises a mix of people, including those with chronic health problems and psycho-social issues [2,14,19,21]. Despite their demographic heterogeneity, studies of frequent ED users do show that they are a vulnerable group [14,16]. For example, they are known to have complex care needs [2,9-12,14,22-24], suffer a higher incidence of mental illness [13], injury [25], morbidity and mortality [12,13], and experience greater social disadvantage [9,10,12,14,21] and homelessness [20,24] than infrequent ED users.

Multiple users are defined by a range of time intervals that range from 48 hours to 120 days [26-31] Patients that return to the ED within 48 to 72 hours have a high risk of error in diagnosis [27]. Specific focus has been placed on elderly patients who have a high rate of return visits to the ED due to their advanced age, isolation, lack of support and poor health [31-35]. These findings are supported by a systematic review conducted by McCusker et al [36] utilising Andersen's behavioural model of service to determinate key factors of ED utilisation.

Furthermore, what constitutes appropriate ED use has not been well defined [8,37], and ED re-presentation has multiple time frames. For example, definitions of frequent use vary from three [38], to twelve visits per year [2,37,39-43]. In contrast, hospital re-admission has been defined as any admission occurring 28 days from discharge, but there is still a lack of agreement about the choice of an appropriate interval [44,45]. Research has determined risk factors associated with hospital re-admission [2,12,19,44,45], and risk factors of return visits to ED for the elderly [28-34,36]. Information on risk factors for re-presentation to the ED focus on quality of care issues such as deficiency in medical management [26,27,46,47]. In addition to counting overall numbers of ED visits per patient per annum, identifying people who re-present to a health service within 28 days is another way of measuring service use. The latter approach to analysis is important because it can capture those people who return to the ED for further treatment within a discrete time frame. Measuring ED service use in this way has the potential to provide a better understanding of frequent ED users and their socio-demographic and clinical characteristics. Indeed, recent research suggests that frequent ED visits may not simply be a reflection of a system unable to properly treat an illness or injury, or a lack of primary care, but rather, a function of complex, interrelated clinical, psycho-social and patient factors [23,43].

The aim of this study was to describe the characteristics of people who re-present to the ED, and identify what factors differentiate these people from those who do not re-present.

## Methods

### Study Design

We examined 24 months of data for all attendances at one ED. The definition of re-presentation was established as attendance at the same ED within 28 days from discharge date from hospital (either from the ED or inpatient unit). This definition of re-presentation is congruent with that of hospital re-admission, which is also defined as occurring within 28 days of discharge.

We used a cohort design to extract and retrospectively analyze contemporaneously collected information from a database of ED presentations to one inner-city ED over a 24-month time frame. The 24-month time frame allowed for the possibility that seasonal differences might influence ED attendance rates. Since October 1995, all publicly-funded EDs within the jurisdiction where this study was conducted, are required to provide standardized information about ED presentations to the State Government through the Emergency Minimum Dataset (EMD) [48]. At the study site, reporting to the EMD is facilitated by the Patient Administration System (PAS), which includes clinical data collected for the use of the service. Data collection practices have been refined to be practical from both a clinical and an organizational perspective.

The study was approved by the Human Research Ethics Committee (HREC) of the health service in which it was conducted. The protocol was subsequently registered as a project with external approval by the collaborating university HREC.

### Setting

The hospital in which this study was carried out is located in a large Australian city with a population of 3.5 million people. The hospital receives its income from State and Federal Government funding and is located within 5 km of the central business district. It is a major teaching hospital, with an ED attendance rate of approximately 32,000 per annum.

### Selection of Participants

Participants included all patients who attended the ED during the study period (January 1, 2003 – December 31, 2004). The only exclusion criteria for the study were patients who had a discharge outcome of death. We considered the inclusion of admitted patients a key factor in the study design since admitted patients often have complex healthcare needs that have implications for discharge planning. Those presentations in December 2004 that had subsequent re-presentation in January 2005 were identified and included in the study. Patient data were retrospectively accessed for analysis from the PAS.

### Methods of Measurement

Data in the PAS contain a unique patient identifier code (unit record number) and detailed demographic and clinical information for each ED presentation. In this study the unit record number was used to classify patients into two primary groups; re-presentation to the ED within 28 days of discharge from hospital or no re-presentation. Missing data from PAS were identified when frequencies showed either inconsistent results or missing information. For analysis, missing data were entered into a category labeled as "unknown".

A standardized data abstraction tool was devised by identifying the key variables and their defined categories within the EMD and replicating this information into a Microsoft Access program (Version 2003, Redmond, Washington). The variables and defining categories that were used in the EMD are displayed in Tables 1, 2, 3, and 4.

**Table 1:** Demographic characteristics of all patients who presented to the emergency department from 1st January 2003 to 31st December 2004 (n = 40,942).

**Variable**	**No**	**%**
**Age (years)**		
<= 25	8745	21.4
26 to 35	9297	22.7
36 to 45	5527	13.5
46 to 55	4483	10.9
56 to 65	3955	9.7
>65	8935	21.8
**Gender**		
Male	22391	54.7
Female	18551	45.3
**Country of Birth**		
Oceania^1^	25187	61.5
Europe	8945	21.8
Asia	4349	10.6
Africa	773	1.9
**Primary Language**		
English	36517	89.2
Italian	1158	2.8
Greek	1009	2.5
Vietnamese	428	1.0
**Aboriginality**^2^		
Yes	558	1.4
No	40384	98.6
**Marital Status**		
Single	20025	48.9
Divorced/Separated	6040	14.8
Married/Defacto	13961	34.1
Unknown	916	2.2
**Interpreter**		
Yes	3498	8.5
No	37444	91.5
**Pensioner**^3^		
Yes	13309	32.5
No	27633	67.5
**Homeless**		
Yes	1595	3.9
No	39347	96.1
**Religion**		
Religious Belief	24168	59.0
None	13449	32.8
Unknown	3325	8.1

Note. 1. Oceania includes: Australia, New Zealand and surrounding islands.  2. Aboriginality is defined as the indigenous population. 3. Pension Card  entitlements include: age (65 and 0ver) and disability.

**Table 2:** Visit characteristics of all patient visits to the emergency department from 1st January 2003 to 31st December 2004 (n = 64,147).

**Variable**	**No**	**%**		
**Arrival Transport**				
Ambulance	21190	33		
Police	1135	1.8		
Car	7231	11.3		
Public Transport	4201	6.5		
Unknown	30390	47.4		
**Australasian Triage Code**				
1 (seen immediately)	863	1.3		
2 (seen within 10 minutes)	5830	9.1		
3 (seen within 30 minutes)	25344	39.5		
4 (seen within 60 minutes)	27229	42.4		
5 (seen within 120 minutes).	4881	7.6		
**Season**				
Summer	16367	25.5		
Autumn	16044	25.0		
Winter	15559	24.3		
Spring	16177	25.2		
**Shift**				
AM shift (0726–1425)	23142	36.1		
PM shift (1426–2135)	24620	38.4		
ND shift (2136–0725)	16385	25.5		
**After Hours (1701–0859)**				
Yes	34154	53.2		
No	29993	46.8		
**Attend Code(Sent in by)**				
Self or Family	45593	71.1		
Other	8569	13.4		
GP	5071	7.9		
Nursing Home	252	0.4		
Other Institution	1510	2.4		
**Presenting Complaint**				
Injury	11841	18.5		
Mental Illness	2980	4.6		
Drug Misuse	1119	1.7		
Medical	45771	71.4		
Other	2436	3.8		
**Left Own Risk**				
Yes	3372	5.3		
No	60775	94.7		
**Discharge Outcome**				
Admit	15898	24.8		
Transfer	1708	2.7		
Home	42667	66.5		
Nursing Home	201	0.3		
Return to Ward	303	0.5		

**Variables**	**Min**	**Max**	**Mean (SD)**	**Median**

**Time to be Seen (Minutes)**	0.0	852.7	46.5(58.7)	25.1
**Inpatient Length of Stay (Days)**	0.04	191.2	7.0(10.2)	4.1
**ED Length of Stay (Hours)**	0.0	96.0	4.6(4.8)	3.4

**Table 3:** Demographic characteristics of all patients who attended the emergency department by housing status from 1st January 2003 to 31st December 2004 (n = 40,942).

**Demographic Variable**	**Housing Status**		
	**Homeless (n = 1,595)**	**Non-homeless (n = 39,347)**	**p value**

Indigenous	106 (6.6%)	452 (1.1%)	<0.001
Single	1145 (71.8%)	18880 (48.0%)	<0.001
Interpreter	55 (3.4%)	3443 (8.8%)	<0.001
Pensioner	1017 (63.8%)	12292 (31.2%)	<0.001
Male	1186 (74.4%)	21205 (53.9%)	<0.001

Note. Chi-Square test undertaken of the hypothesis of equal proportions. Indigenous: includes Aboriginal or Torres Strait Islander. Marital Status: involves proportion with a single status. Interpreter: indicated if requested in the Patient Administration System for communication purposes. Pensioner: involves recipient of a government pension.

**Table 4:** Percentage of visits resulting in re-presentation to the emergency department and adjusted odds ratios for the final model by significant patient and emergency department visit characteristics 1 January 2003 to 31 December 2004.

**Explanatory Variable**	**All Visits % (n = 64,147)**	**All Patients % (n = 40,942)**	**% Predicted by Model**	**Adjusted Odds Ratio (95% CI)**
**Total Re-presentation**	18.0 (n = 11559)	6.9 (n = 5894)		
**Patient Characteristics**				
				
**Homeless**				
No	14.5 (n = 57458)	6.3 (n = 39347)	11.8	Reference
Yes	47.8 (n = 6689)	22.7 (n = 1595)	34.7	3.09 (2.83, 3.36)
**Pensioner**				
No	11.6 (n = 35487)	5.0 (n = 27633)	9.5	Reference
Yes	25.9 (n = 28660)	10.9 (n = 13309)	18.5	1.73 (1.63, 1.84)
**Gender**				
Female	16.2 (n = 28328)	6.3 (n = 18551)	11.9	Reference
Male	19.5 (n = 35819)	7.4 (n = 22391)	13.7	1.16 (1.10, 1.22)
**Age**				
25 years and under	11.5 (n = 11162)	4.8 (n = 8745)	9.2	Reference
>25 years	19.4 (n = 52985)	7.5 (n = 32197)	13.8	1.21 (1.12, 1.32)
**Marital Status**				
Single	18.0 (n = 30321)	6.4 (n = 20025)	12.4	Reference
No Longer Married	22.6 (n = 11709)	9.3 (n = 6040)	16.2	1.13 (1.04, 1.22)
Married/de facto	16.3 (n = 21058)	6.8 (n = 13961)	12.4	1.04 (0.97, 1.11)
Unknown	6.9 (n = 1059)	2.9 (n = 916)	5.6	0.43 (0.33, 0.56)
**Interpreter Required**				
No	17.6 (n = 57180)	6.3 (n = 37444)	12.5	Reference
Yes	21.2 (n = 6967)	9.4 (n = 3498)	16.6	1.13 (1.04, 1.22)
**ED Visit Characteristics After Hours**				
0900 – 1700	17.3 (n = 29993)		12.5	Reference
1701 – 0859	18.6 (n = 34154)		13.3	1.11 (1.06, 1.17)
**Attendance Source**				
Other Institutions (Hospitals/Correctional)	8.9 (n = 1510)		7.7	Reference
Community Nurse	20.1 (n = 2861)		12.4	1.31 (1.06, 1.63)
Hospital Service (Out/Inpatient, ED Review)	26.4 (n = 269)		14.4	1.83 (1.28, 2.62)
Crisis Assessment Team	36.4 (n = 22)		24.0	2.97 (1.05, 8.41)
Self/Family/Friends	18.3 (n = 45593)		12.7	1.46 (1.21, 1.77)
General Practitioner	15.7 (n = 5071)		13.1	1.46 (1.19, 1.80)
Nursing Home	15.9 (n = 252)		13.2	1.09 (0.72, 1.66)
Other	18.5 (n = 8569)		12.6	1.42 (1.16, 1.73)
**Discharge Outcome**				
Home	17.9 (n = 42667)		13.0	Reference
Transfer Other Facility	8.1 (n = 1708)		6.3	0.43 (0.36, 0.53)
Left Unseen	25.1 (n = 2654)		15.8	1.19 (1.06, 1.33)
Left Part Treatment	33.0 (n = 716)		21.2	1.64 (1.36, 1.99)
Admit	17.6 (n = 15898)		12.8	0.85 (0.81, 0.90)
Residential Care Facility	31.8 (n = 201)		21.9	1.46 (1.03, 2.06)
Return to Ward	32.3 (n = 303)		18.4	1.16 (0.88, 1.53)
**Presenting Complaint**				
Injury	11.3 (n = 11841)		9.4	Reference
Mental Illness	28.5 (n = 2980)		18.5	1.65 (1.47, 1.86)
Drug Misuse	36.6 (n = 1119)		14.4	1.20 (0.99, 1.46)
Medical/Other	18.6 (n = 48207)		13.6	1.38 (1.28, 1.48)
**ED Length of Stay (Hours)**				1.01 (1.01, 1.02)

For this study, homelessness comprised various living arrangements such as residing in a public place, a boarding house/hostel, a homeless shelter or having no fixed address. For each ED presentation at the study site, information about housing status was captured in the PAS by the variable, usual accommodation. To ensure the accuracy of this variable the patients identified as homeless had further examination of their medical records to confirm and identify the level of homelessness.

For the purpose of analysis, Chamberlain's [49] classification of housing status was used to create five categories. Primary homelessness included people living on the streets or in squats. Secondary homelessness included people living in crisis accommodation. Tertiary homelessness included people living in boarding houses. Marginalized housing included people residing in public housing requiring rental assistance or those with other unstable rental arrangements. Stable housing included people living in permanent accommodation.

### Data Collection and Processing

Due to the volume of data generated by an electronic report over the 24-month period, three abstractors were used to collate the data for those patients who were homeless. Training was conducted over two days and review of abstractor accuracy was performed on a daily basis. One abstractor took the position of leader in the data collection process and examined 80% of all data collected. All abstractors were blinded to the etiologic relationships being investigated in the study.

The abstractors conducted an independent review of the first 20 medical records for the purpose of determining inter-rater reliability. Inter-rater reliability was examined in the coding of the homeless variable. The percentage of agreement for the category homeless or not homeless was 96.7% for 3 raters, 20 cases and 2 categories. The percentage of agreement for the level of homelessness was 73.3% for 3 raters, 20 cases and 5 categories. In 12 of the 20 cases all 3 raters agreed with the 5 categories and with the other 8 cases 2 of the raters agreed. The level of homelessness was the only variable that required an assessment from the abstractors. It was not difficult to assess that someone was homeless but there was a degree of difficulty in determining the level of homelessness.

### Primary Data Analysis

All data were entered into a Microsoft Access database and were subsequently analyzed using SPSS, version 14.0 (Chicago, IL). The ED presentation data (*n *= 64,147) were examined by constructing a frequency cross-tabulation of re-presentation (yes/no) against each categorical explanatory variable (factor), and by constructing a table of means for each continuous explanatory variable (covariate). This information containing ED presentation data was aggregated into a patient dataset (*n *= 40,942) that contained the percentage of attendances resulting in re-presentation for each patient. The patient data were examined by constructing a table of the mean percentage for each factor. Because of the unequal numbers of presentations per patient (min = 1: max = 152), the percentage of all visits resulting in re-presentation was larger than the mean percentage calculated across patients, for each category of each factor. For example, if we consider patients who were pensioners (*n *= 28,660), 25.9% of their visits resulted in re-presentation, but the mean percentage calculated across these patients was 10.9%. Because the probability of re-presentation is correlated between visits within a patient, neither of the above figures is an appropriate estimate of the true percentage, *p*. The percentage of all visits is an overestimate of *p*, because it gives equal weight to each visit, and so is inflated by patients with a large number of visits. The mean percentage across patients is an underestimate of *p*, because it gives equal weight to each patient, and so effectively ignores the additional information provided by the multiple visits of many patients.

We dealt with the potential problem of underestimation or overestimation of re-presentation by developing a generalized linear mixed model (GLMM) with a binomial distribution. This model uses all the data to estimate the correlation between visits within a patient, and gives an estimate of *p *which gives an appropriate effective weighting to visits and patients. In the example involving pensioners, the estimate is 17.5%, which is between the two percentages mentioned. For this example, the estimate is about half-way between but in other cases it can be much more toward one side, depending on the correlation for the group of interest. The GLMM used for this study was a logistic regression model with the patient variable fitted as a random effect to account for repeated presentations. The fitting of the model was performed using GenStat (8^th ^edition, VSN International, UK).

For each explanatory variable on its own, a GLMM was fitted with re-presentation (yes/no) as the response variable. The *P*-value for testing the hypothesis of no effect was determined by comparing the Wald statistic with the appropriate chi-square distribution. Variables for which the *P*-value was greater than 0.01 were excluded from further model fitting. All the remaining explanatory variables were entered into a backwards stepwise procedure, in which the least significant variable at each stage was progressively removed from the model, until all variables still in the model were significant at the 0.01 level. The 0.01 cut-off was chosen rather than the more common value of 0.05 because the large number of observations meant that a small *P*-value could arise from an inconsequential effect size.

The estimated parameters and standard errors from the final model were used to calculate adjusted odds ratio (OR) and associated 95% confidence intervals for each explanatory variable. There were twelve of them – seven related to patient characteristics, and five related to ED visit characteristics. For explanatory variables not in the final model, unadjusted OR and 95% confidence intervals were calculated.

Although the housing status comprised five categories, there were no significant differences between the four homeless levels in terms of their re-presentation percentages, so in the final model they were combined into a single category. However, because of the importance of this variable to our study, a separate model was fitted with all five categories, and adjusted OR and confidence intervals calculated.

## Results

A total of 64,147 emergency department visits by 40,942 individuals occurred between 1 January 2003 and 31 December 2004 inclusive. The mean ED length of stay for the cohort was 4.60 hours (SD = 4.78) and the mean in-patient length of stay was 7.03 days (SD = 10.22). The number of presentations varied from 1 to 152 per patient with the mean number being 1.75. Of the study group, 54.7% (n = 22,391) were male, 48.9% (n = 20,025) were single and 32.5% (n = 13,309) were receiving a pension and 1.4% (n = 558) were indigenous. The mean age was 45 (SD = 21), median 40, with the range of age from 1 to 107. The percentage of indigenous people re-presenting to the ED was higher than the percentage in the general population, since 0.6% (n = 29,683) of people residing in the state are of an indigenous background [50]. Those with a European or Asian country of birth made up 32.4% (n = 13,294) of the study group with 26.3% (n = 3,498) of this population requiring an interpreter on presentation to the ED. Australia has a relatively large proportion of immigrants making up 24% of the population with a quarter of the immigrants born in Asia [51].

Table 1 shows the demographic characteristics of patients who presented to the ED for the study period. Homeless people accounted for 3.9% (n = 1,595) of the total study population and 10.4% (6,689) of all presentations and 27.7% (n = 3,199) of all re-presentations. Table 2 shows the visit characteristics of patients who presented to the ED during the two-year period. Those patients who presented after hours represented 53.2% (n = 34,254) of the total presentations. Patients who left at own risk (n = 3,372) represented 5.3% of all the presentations. Table 3 shows the demographic characteristics of all patients who attended the ED during the study period by housing status (homeless/non homeless) that are significantly different with a p value < 0.001. The fact that homeless people had a significantly higher incidence of being on the pension and a significantly lower incidence of accessing an interpreter reveals additional issues of vulnerability associated with homelessness. Due to the limited information in medical records it was not possible to explore the utilisation of interpreting services.

The rate of re-presentation for the total population was 18.0% (n = 11,559) of visits and 14.4% (n = 5,894) of patients. The mean time to re-presentation was 7.9 days and the median was 6.0 days. More than half of all re-presentations occurred within one week of discharge from the ED (60.8%; n = 6,873), and more than three-quarters re-presented within two weeks of the initial presentation (80.9%; n = 9,151). Of those patients who re-presented within one week, 66.4% (n = 7,506) were initially discharged home and 55.3% (n = 6,253) attended the ED after business hours. A small group re-presented while an inpatient (n = 251) and they were excluded from these figures.

The patient variables that significantly influenced re-presentation of patients to the ED are shown in Table 4. Most notably, housing status (being homeless) and source of income (being a recipient of a government pension) were associated with increased odds of re-presentation (adjusted OR 3.09; 95% CI, 2.86 to 3.36; adjusted OR 1.73; 95% CI, 1.63 to 1.84 respectively). In addition, our analysis showed that a presenting complaint of mental illness increased the odds of re-presentation by 1.65 times compared to injury (95% CI, 1.47 to 1.86). Where patients left part-way through treatment they had 1.64 times greater odds of re-presenting than if they completed treatment and were discharged home (95% CI, 1.36 to 1.99). Being discharged to a residential care facility increased the odds of re-presentation by 1.46 times when compared to being discharged home (95% CI, 1.03 to 2.06).

While housing status (homeless) was highly significant in determining the likelihood of re-presentation to the ED, within the levels of homelessness, there were no significant differences between the primary, secondary, tertiary and marginalized housing groups. Table 5 shows the number and percentage of visits resulting in re-presentation and adjusted odds ratios by housing status.

**Table 5:** Percentage of visits and of patients resulting in re-presentation and adjusted odds ratios by housing status.

**Explanatory Variable**	**All Visits (n = 64,147)**	**All Patients (n = 40,942)**	**% Predicted by Model**	**Adjusted Odds Ratio (95%CI)**
**Housing status**				
Primary homeless	43.8 (n = 1097)	19.7 (n = 369)	32.0	3.54 (3.00, 4.21)
Secondary homeless	56.7 (n = 1279)	24.8 (n = 263)	38.6	4.68 (4.00, 5.62)
Tertiary homeless	43.5 (n = 1858)	23.3 (n = 441)	34.3	3.90 (3.38, 4.49)
Marginalized housing	48.3 (n = 2455)	21.8 (n = 522)	34.5	3.93 (3.45, 4.48)
Stable housing	14.5 (n = 57458)	5.9 (n = 39347)	11.8	Reference

Note. Primary homeless: people living on the streets or in squats. Secondary homeless: people living in crisis accommodation. Tertiary homeless: people living in boarding houses. Marginalized housing: those people residing in public housing requiring rental assistance or those with other unstable rental arrangements. Stable, non homeless: people living in permanent accommodation.

## Discussion

Emergency re-presentations were found to account for almost one-fifth (17.4%) of all ED visits. This proportion is slightly higher than overall hospital re-admission rates, which have been reported to range from 5 to 14 percent in general populations [52-54] and from 12 to 16 percent in geriatric populations [44,55,56].

The pattern of ED re-presentation described in this study showed that rates clustered around one week after the first visit and fell rapidly thereafter. This result is also similar to work on patterns of hospital re-admission [44], but it is not possible to tell whether early ED re-presentations are due to factors that are amenable to targeted interventions or the result of other non-preventable factors including the normal progression of disease.

Our study found that the characteristics influencing re-presentation to the ED included a range of socio-economic factors; in particular housing status and receiving a government pension were identified as significant risk factors for re-presentation. These findings are consistent with work on frequent ED use [5,10,13,16,18,19,42] but are new to our understanding of factors influencing ED re-presentation.

While a homeless housing status was highly significant in determining the likelihood of re-presentation to the ED, within the levels of homelessness there was no significant difference between the primary, secondary, tertiary and marginalized housing categories. This finding has important clinical implications in trying to identify patients at risk of re-presentation. It may be easier to identify someone living on the streets, but those patients who have an address but fit into a marginalized housing category are not so easily identified by hospital staff, even though they are equally at risk.

To date interventions designed to decrease ED attendances have involved two main strategies; increasing referrals to alternative sources of care, such as general practices [57-59], and referral to care coordination programs and community based support services for those people at risk [23]. However, evaluation of these strategies has produced mixed results[23,60]. For example, increasing the number and availability of general practice clinics, especially in close proximity to the ED, is one approach that has been suggested to decrease ED demand [59]. This approach has, by in large, not been successful because many ED patients, even those classified as non-urgent, are more complex and require longer consultation times than general practice patients [59]. In addition, people who attend EDs with less urgent problems have higher hospital admission rates than those who attend general practices [61].

Care co-ordination aims to decrease ED re-presentation rates [23]. Using this approach, patients are screened to determine their likelihood of re-presenting to hospital at ED triage. Risk factors for re-presentation commonly include; age >65 years, living alone, significant care responsibilities, receiving community services and self care problems [59]. Diagnostic categories for heart failure, chronic obstructive pulmonary disease (COPD) and asthma have been found to increase the risk of hospital re-admission and high levels of deprivation, that is low socio-economic status, is significantly and independently associated with increased odds of emergency medical re-admission [45].

This research was conducted at a single inner-city ED in Australia, thus these findings cannot be generalized to other locations. However, since we used data elements mandated for reporting to the EMD, this work has the potential to be replicated in other Australian EDs and internationally. Although there is potential for national replication the Australian health care system does differ substantially from other countries and therefore it may not be possible to directly compare rates and patterns of ED re-presentation to those found elsewhere in the world.

The study only had data for presentations to the targeted hospital and it was not possible to identify those patients who may have presented to other hospitals after attending the study hospital. Patients who died as a discharge outcome were excluded from the study but the data on subsequent deaths occurring after discharge were unreliable due to incomplete data. These individuals were not known and therefore not excluded.

A further factor limiting comparison between ED re-presentation rates is the lack of information currently collected on housing status by EDs. Our study could identify housing status as a factor significantly influencing re-presentation rates since this information was available on the hospital ED PAS database and confirmed by review of patients' medical records. The data collection of other EDs through the EMD does not routinely include this crucial information. One possible reason for not including this information in the EMD may relate to the different ways in which homelessness is understood and defined [62].

Although the study was a retrospective analysis, we included the total population for a defined period of time and aside from death, there were no exclusions. Since the data used were mandated for reporting through the EMD the data set was relatively complete and objective.

## Conclusion

A definition for ED re-presentation and housing status has clinical relevance to assist in early recognition of risk factors and targeting specific resources. The fact that homeless people have a significantly higher incidence of being on the pension and a significantly lower incidence of accessing an interpreter reveals additional issues of vulnerability associated with homelessness.

Many people present to the ED after hours and this makes accessing and referral to services other than those of a medical nature more difficult. More than 50% of patients in our study who re-presented in the first week did so after hours. This finding has implications for referral practices. Service provision for patients who not only have a medical condition but are homeless is complex but unless a hospital system develops strategies for early recognition and referral with a broader definition of homelessness, patients will continue to re-present with ongoing issues that may not be confined to their medical condition.

## Competing interests

There have been no previous publications of this study and there is no perceived conflict of interest or copyright constraints. The authors declare that they have no competing interests.

## Authors' contributions

EM, MG and AD conceived the study, designed the method, and obtained research funding. EM and MG are the head supervisors, AD was a co-supervisor and a clinical expert and GM is the PhD candidate. EM, MG and AD supervised the conduct of the study and data collection. GM undertook collection and overseeing data abstraction, development of abstractor tools and analysis, including quality control. GH provided statistical advice on study design and supervised analysis of data. MG and GM drafted the manuscript, and all authors contributed substantially to its revision and approved the final manuscript. GM takes responsibility for the paper as a whole. All authors have read and approved the final manuscript.

## Pre-publication history

The pre-publication history for this paper can be accessed here:


